# Alterations in cortical surface morphometry in fibromyalgia: a multi-parametric study

**DOI:** 10.3389/fpsyg.2026.1813590

**Published:** 2026-05-25

**Authors:** Jiancheng Hou, Changan Sun

**Affiliations:** 1 Research Center for Cross-Straits Cultural Development, Fujian Normal University, Fuzhou, China; 2 Department of Radiology, School of Medicine and Public Health, University of Wisconsin-Madison, Madison, WI, United States; 3 School of Education, Suzhou University of Science and Technology, Suzhou, China

**Keywords:** fibromyalgia, surface-based morphometry, multi-parametrics, pain, clinical measures

## Abstract

**Introduction:**

Fibromyalgia (FM) is a chronic pain disorder with unclear neuroanatomical basis, as previous studies largely relied on volumetric measures insensitive to cortical surface morphology. This multi-parametric surface-based morphometry study examined cortical thickness (CT), fractal dimension (FD), gyrification index (GI), and sulcal depth (SD) in FM.

**Methods:**

Thirty-three female FM patients and thirty-three age-matched female healthy controls (HC) underwent T1-weighted MRI. Group differences were assessed using non-parametric permutation testing (TFCE, *p* < .05). Correlations with clinical measures (depression, anxiety, alexithymia, pain, disease impact) were explored.

**Results:**

FM patients showed widespread bidirectional alterations across all four metrics. CT was greater in the right posterior cingulate but lower in the left superior frontal gyrus (SFG), superior parietal lobule (SPL), caudal anterior cingulate (cACC), and right medial orbitofrontal cortex. FD was greater in sensorimotor regions (bilateral postcentral gyri, right precentral gyrus) but lower in visual/temporal areas (bilateral fusiform, left superior temporal gyrus). GI was greater in bilateral SPL and left SFG but lower in the right precuneus and precentral gyrus. SD was lower in bilateral supramarginal gyri and right inferior parietal lobule but greater in the left pars triangularis and right caudal middle frontal gyrus. Clinically, FM patients showed significantly higher depression, anxiety, and alexithymia. After correction, CT in the left cACC remained positively correlated with depression in FM.

**Discussion:**

FM is associated with a multifaceted cortical phenotype involving pain processing, emotion regulation, and self-referential cognition regions. These structural alterations, partly correlated with clinical symptoms, provide a robust framework complementing existing functional models of FM.

## 1 Introduction

Fibromyalgia (FM) is a complex and debilitating chronic pain disorder that predominantly affects women, with a global prevalence estimated to be significantly higher in females than in males. Clinically, FM is characterized by widespread, persistent pain, often accompanied by a constellation of other debilitating symptoms, including profound fatigue, unrefreshing sleep, cognitive disturbances (“fibro-fog”), and marked emotional distress (Kravitz and Katz, 2015). The high prevalence of psychiatric comorbidities, particularly depression and anxiety, underscores the intricate relationship between physical and emotional dysfunction in this condition (Galvez-Sanchez et al., 2019; Rahangdale and Ferraro, 2025). The lifetime prevalence of major depressive disorder in FM patients is estimated to be over 50%, with anxiety disorders affecting up to one-third of individuals (Oliveria Neto et al., 2025). This affective imbalance—characterized by diminished positive affect and heightened negative affect—is not merely a secondary consequence of living with chronic pain; it is intrinsically linked to pain intensity, overall symptom severity, and reduced quality of life (Rost et al., 2021; Stroman et al., 2025; Zautra et al., 2005).

The prevailing pathophysiological model for FM is one of central sensitization, a condition in which the central nervous system (CNS) amplifies sensory input, leading to hypersensitivity to painful (hyperalgesia) and non-painful (allodynia) stimuli (Jurado-Priego et al., 2024). This aberrant processing is thought to be driven by complex neurobiological alterations, including neuro-inflammation and imbalances in neurotransmitter systems (Sluka and Clauw, 2016). Neuroimaging has been instrumental in advancing our understanding of these CNS abnormalities, revealing that FM is not a disease of the peripheral tissues but one of altered brain structure, function, and connectivity (Jensen et al., 2013; Napadow and Harris, 2014).

Functional magnetic resonance imaging (fMRI) studies have consistently demonstrated aberrant brain activity and functional connectivity (FC) in FM patients, particularly within the neural networks subserving pain modulation, salience detection, and emotion processing (Stroman et al., 2025). The “pain matrix,” a distributed network of brain regions including the thalamus, insula, anterior cingulate cortex, and prefrontal cortices, shows heightened responses to both painful and non-painful stimuli in FM (Ackermann et al., 2025; Staud, 2011). More recently, research has focused on the interplay between pain and emotion. As highlighted in the study by Balducci et al. (2024), FM patients exhibited abnormal brain activation and connectivity during the processing of emotional stimuli. Their fMRI findings revealed hyperactivation of the left superior lateral occipital cortex during emotion processing, irrespective of valence, in patients compared to healthy controls. This hyperactivation was also positively correlated with the severity of depression and anxiety, suggesting that visual attention and pain modulation networks may be recruited differently in FM during emotional experiences. Furthermore, Balducci et al. identified a valence-dependent disruption in the functional connectivity of the left pregenual anterior cingulate cortex (pACC)—a critical hub for the integration of affective and sensory information—with sensorimotor and opercular regions. Specifically, they observed greater pACC connectivity during positive emotion processing and lower connectivity during negative emotion processing in the FM group. These findings provided crucial evidence that the neural circuits governing emotional experience are fundamentally altered in FM, potentially explaining the high prevalence of psychopathological symptoms and the strong affective component of pain.

While task-based fMRI studies like that of Balducci et al. shed light on the dynamic functional abnormalities underlying specific cognitive and affective processes, structural MRI offers a complementary perspective by characterizing the underlying neuroanatomical architecture. Traditional volumetric analyses, such as voxel-based morphometry (VBM), have been widely used to investigate gray matter (GM) changes in FM, with studies reporting both increases and decreases in GM volume in regions such as the cingulate cortex, insula, and prefrontal cortex; the less volume in regions such as the cingulate cortex and prefrontal cortex reflects alterations in emotional and cognitive processing networks (Lin et al., 2016; Xin et al., 2023), while the increased volume in sensorimotor areas may be associated with chronic nociceptive input, although causal interpretations require longitudinal data (Xin et al., 2023). Although these structural alterations provide a neuroanatomical foundation for the diverse clinical symptoms of FM, including pain hypersensitivity, emotional disturbances, and cognitive dysfunction (Kravitz and Katz, 2015), the volumetric measures have some limitations: they are relatively insensitive to the complex folding of the cortical surface, which can introduce regional biases and reduce statistical power. Moreover, the inherent MRI intensity contrast between GM and white matter (WM) can lead to an underestimation of true morphological differences (Bermudez et al., 2009). Consequently, observed volumetric changes may actually be driven by more specific aspects of cortical surface morphometry, such as cortical thickness (CT), fractal dimension (FD), gyrification index (GI), and sulcal depth (SD).

These surface-based metrics can provide a more nuanced and fine-grained characterization of cortical anatomy. CT measures the distance between the pial and white matter surfaces, reflecting the density and arrangement of neurons, and is a sensitive index of GM integrity (Hirakawa et al., 2016; Seiger et al., 2018). FD quantifies the complexity of the cortical folding pattern across multiple spatial scales, capturing irregularities in shape that may reflect underlying disruptions in neuronal organization (Di Ieva et al., 2015; Di Ieva et al., 2014; Madan and Kensinger, 2017; Yotter et al., 2011b). FD has been shown to be sensitive to GM alterations that are not captured by volume or thickness alone and may be less susceptible to certain confounds, such as participant’s sex (Chen et al., 2020; Madan and Kensinger, 2017; Zhang et al., 2008). GI assesses the degree of local cortical folding, which is established primarily during neurodevelopment and can offer insights into the developmental origins of neurological and psychiatric conditions (Li Y. et al., 2021). Finally, SD measures the Euclidean distance from the cortical surface to a reference hull, reflecting the profoundness of sulci (Li Y. et al., 2021; Yun et al., 2013). It is thought to be influenced by the combined effects of GM and WM changes and is particularly sensitive to the complex folding of the cerebral surface (Im et al., 2008; Jin et al., 2018; Kim et al., 2008; Kochunov et al., 2008).

Despite the wealth of functional neuroimaging studies in FM, investigations into alterations in cortical surface morphometry have been relatively sparse and have yielded inconsistent findings. Some studies have reported regional cortical thinning in FM, for example in the cingulate and parahippocampal gyri (Oliveria Neto et al., 2025), while others have found thickening in pain-related regions like the somatosensory cortex (Xin et al., 2023). Similarly, studies on gyrification have reported both hypo- and hyper-gyrification in different cortical areas, possibly reflecting a combination of developmental traits and disease-related plasticity (Tu et al., 2022). To date, no study has comprehensively examined changes in a multi-parametric set of surface-based morphometry (SBM) measures—specifically CT, FD, GI, and SD—in the same cohort of FM patients. Each of these indices captures a unique aspect of cortical structure, and together, they can mutually disambiguate and complement each other’s weaknesses to provide a more complete picture of the neuroanatomical alterations associated with the disorder. For instance, while CT provides information about laminar integrity, FD and GI offer insights into the complexity and pattern of cortical folding, and SD reflects the geometry of sulci (Tu et al., 2022). Integrating these measures could reveal a more cohesive and detailed account of the cortical changes that may underpin the complex symptomatology of FM, from pain perception to emotional dysregulation.

Therefore, the current study aimed to fill this gap by employing a comprehensive surface-based morphometry approach to investigate alterations in cortical structure in female patients with fibromyalgia compared to age-matched female healthy controls. By utilizing the publicly available dataset described by Balducci et al., we analyzed CT, FD, GI, and SD to explore the cortical surface differences between the groups. We hypothesized that patients with FM would exhibit significant alterations in these cortical metrics in brain regions critically involved in pain processing, sensory integration, and emotion regulation, such as the cingulate cortex, insula, prefrontal cortex, and somatosensory areas. Furthermore, we would explore the relationship between these structural alterations and clinical measures of pain, depression, anxiety, and alexithymia to better understand the structural brain basis of the heterogeneous clinical presentation of fibromyalgia.

## 2 Methods

### 2.1 Participants

The participants’ information, and their anatomical T1-weighted imaging dataset, were obtained from a public dataset via OpenNeuro with accession number ds004144.^1^ There were 33 female FM patients (mean age = 41.727 ± 6.094 years old, ranged from 30 to 50 years) and also 33 female healthy controls (HC; mean age = 41.515 ± 6.032 years old, ranged from 29 to 50 years) were selected. No significant age difference was found (*t*_(64)_ = 0.142, *p* = 0.887). Ethical approval was granted by the Research Ethics Committee of the National Institute of Psychiatry “Ramon de la Fuente Muñiz” in Mexico City. The inclusion and exclusion criteria followed those outlined by Balducci et al. (2024).

### 2.2 Clinical measures

Five clinical measures were used. To measure severity of depression and anxiety, the 17-items Hamilton Depression Rating Scale (HAMD) (Ramos-Brieva and Cordero-Villafafila, 1988) and the Hamilton Anxiety Rating Scale (HAMA) (Lobo et al., 2002) were administered. Moreover, alexithymia was measured using the Toronto Alexithymia Scale (TAS), a self-rating scale (Bagby et al., 1994). Furthermore, the Fibromyalgia Impact Questionnaire (FIQ) (Burckhardt et al., 1991) and the McGill Pain Questionnaire (MPQ) (Masedo and Esteve, 2000; Melzack, 1975) were administrated in the FM group to evaluate the severity of fibromyalgia and the characteristics of pain, respectively; no data were collected from the HC group for these measures.

### 2.3 MRI data acquisition

Whole-brain anatomical T1-weighted image data were acquired using a 3.0 T Philips Ingenia Magnetic Resonance Imaging scanner with a 32-channel phased array head coil. The parameters were: TR = 7.7 ms, TE = 3.2 ms, flip angle = 12°, field of view = 256 × 256 mm^2^, matrix = 256 × 256, slice thickness = 1 mm, number of slices = 168, gap = 0 mm.

### 2.4 Data preprocessing

The Computational Anatomy Toolbox (CAT12),^2^ which is a plug-in software based on Statistical Parametric Mapping (SPM12)^3^ and integrated into MATLAB (MathWorks), was used for the T1-weighted MRI data preprocessing. The CAT12 is not only a more precise and accurate analysis of gray matter volume than the previous voxel-based morphometry plug-in toolbox in SPM (Farokhian et al., 2017; Yuksel et al., 2018) but also is fully automated for surface-based analysis (Zhuang et al., 2017). The data preprocessing with CAT12 consisted of bias-field correction, skull-stripping, and alignment to the Montreal Neurological Institute (MNI) structural template to classify gray matter (GM), white matter (WM) and cerebrospinal fluid (CSF), as well as spatial normalization with the Diffeomorphic Anatomical Registration Through Exponentiated Lie Algebra (DARTEL) registration (1.5 mm) (Kurth et al., 2015; Yuksel et al., 2018; Zhuang et al., 2017). Subsequently, we employed a spherical harmonic method (Yotter et al., 2011a) to reparametrize the cortical surface mesh based on an algorithm that reduces area distortions (Yotter et al., 2011c) to repair any topological defects (Chen et al., 2020; Yotter et al., 2011a; Yotter et al., 2011c).

CT was analyzed based on the workflow specified in the study by Dahnke et al. (2013). This algorithm used tissue segmentation and evaluated the WM distance by estimating the distance from the inner GM boundary for each GM voxel. Values at the outer GM boundary in the WM distance map was projected back to the inner GM boundary to generate the GM thickness (Li Y. et al., 2021). Following this, a central surface was created at the 50% level of the percentage position between the WM distance and GM thickness (Li Y. et al., 2021). For the resultant central surface, a topology correction based on spherical harmonics was used to account for topological defects (Li Y. et al., 2021; Yotter et al., 2011a). Moreover, the central surface was reparameterized into a common coordinate system through spherical mapping, and spherical registration employed the volume-based diffeomorphic DARTEL algorithm to the surface (Li Y. et al., 2021). A spatially smoothed 15 mm full width at half maximum (FWHM) Gaussian kernel was used for CT analysis. This smaller kernel was chosen because CT reflects radial laminar organization and typically exhibits relatively smooth spatial gradients; a 15 mm kernel preserves regional specificity while reducing high-frequency noise (Dahnke et al., 2025).

FD estimates cortical fold complexity based on spherical harmonic reconstructions (Li Y. et al., 2021; Yotter et al., 2011a) and was calculated as the slope of a logarithmic plot of surface area versus the maximum l-value, where the maximum l-value is a measure of the bandwidth of frequencies used to reconstruct the surface shape (Li Y. et al., 2021; Yotter et al., 2011b). For FD analysis, a smoothed 20 mm FWHM Gaussian kernel was applied.

Based on the spherical harmonic reconstructions, GI, as an indicator of cortical folding, was calculated as absolute mean curvature (Li Y. et al., 2021; Luders et al., 2006). Mean curvature is an extrinsic surface measure, and it provides information about the change in normal direction along the surface (Li Y. et al., 2021). A smoothed 20 mm FWHM Gaussian kernel was used for GI analysis.

SD measures the depth of sulci and was calculated as the Euclidean distance between the central surface and its convex hull based on the spherical harmonic reconstructions, then transformed with the sqrt function (Li Y. et al., 2021). A smoothed 20 mm FWHM Gaussian kernel was used for SD analysis.

The use of different smoothing kernels for different metrics follows the standard defaults in CAT12 and is justified by the distinct spatial frequency characteristics of each measure. Specifically, FD, GI, and SD capture coarser aspects of cortical folding complexity and sulcal geometry that operate at larger spatial scales and are more sensitive to high-frequency surface undulations; therefore, a larger 20 mm kernel is recommended to stabilize these estimates and improve cross-subject alignment. This practice has been widely adopted in previous multi-parametric surface-based morphometry studies (Li M. et al., 2021b; Tu et al., 2022).

### 2.5 Statistical analysis

All morphometric (CT, FD, GI SD) differences between the FM and HC groups were conducted in CAT12. The age and intracranial volumes were considered as covariates. Group differences were assessed using a non-parametric permutation approach and threshold-free cluster enhancement (TFCE) *p* < 0.05 after 5,000 permutations, which was used for multiple comparison correction (Smith and Nichols, 2009). We focused on these significantly different brain regions with cluster size greater than 100 vertices (cluster size × percentage covered in the specific regions). All results were labeled using the Desikan–Killiany atlas (DK40) (Desikan et al., 2006) to label the cortical regions and were visualized using the CAT12.

For brain regions identified as significantly different between groups, the mean value in significant cluster was extracted through the CAT12 statistics module. These values were then subjected to correlation analyses with each clinical measure using Pearson’s correlation in SPSS (version 23). This approach of using the mean across a cluster is standard practice, as it provides a robust and representative measure of a region’s structural characteristic, while also increasing statistical power (Eickhoff et al., 2016; Poldrack, 2007).

Additionally, for the correlation analyses between cortical metrics and clinical measures, the present study initially reported uncorrected *p*-values with SPSS for exploratory purposes. However, to address concerns regarding multiple comparisons, the present study also subsequently applied Bonferroni correction (the number of tests in the FM or HC group: 4 cortical metrics × 14 regions showing significant group differences × 5 clinical measures = 280 tests; *α* = 0.05 divided by the 280 tests, and corrected *α* = 0.00018) as well as false discovery rate (FDR) correction (*q* < 0.05).

## 3 Results

### 3.1 Clinical measures

The two-group *t*-test results revealed significant differences on multiple clinical measures. Specifically, the FM group exhibited significantly higher scores than the HC group on the HAMD, HAMA, and TAS. In contrast, no significant group differences were observed for the FIQ or MPQ (see Table 1). These results indicate that, compared to healthy controls, the FM group reported substantially higher levels of depression, anxiety, and alexithymia, whereas the two groups did not differ significantly on the overall impact of fibromyalgia or on the self-reported pain characteristics as measured by the MPQ. Notably, the FIQ and MPQ were administered only to the FM group. The values shown for the HC group in these two columns are not actual measurements and are provided only for tabular completeness; no statistical inference is intended or should be drawn.

**Table 1:** Clinical measure differences between FM and HC groups.

Clinical measure	Mean scores		*t*	*p*
	FM	HC		
HAMD	15.567 (6.374)	1.212 (1.933)	12.388^****^	0.000
HAMA	21.546 (6.833)	2.182 (2.430)	15.338^****^	0.000
TAS	59.667 (21.563)	38.909 (11.912)	4.840^****^	0.000
FIQ	3.546 (2.137)	2.697 (2.186)	1.594	0.116
MPQ	1.758 (1.091)	1.727 (0.911)	0.123	0.903

^****^p < 0.001. The FIQ and MPQ were administered only to the FM group. The HC values are placeholders for tabular formatting and do not represent actual data.

### 3.2 Cortical analysis

#### 3.2.1 CT

**Figure 1:**
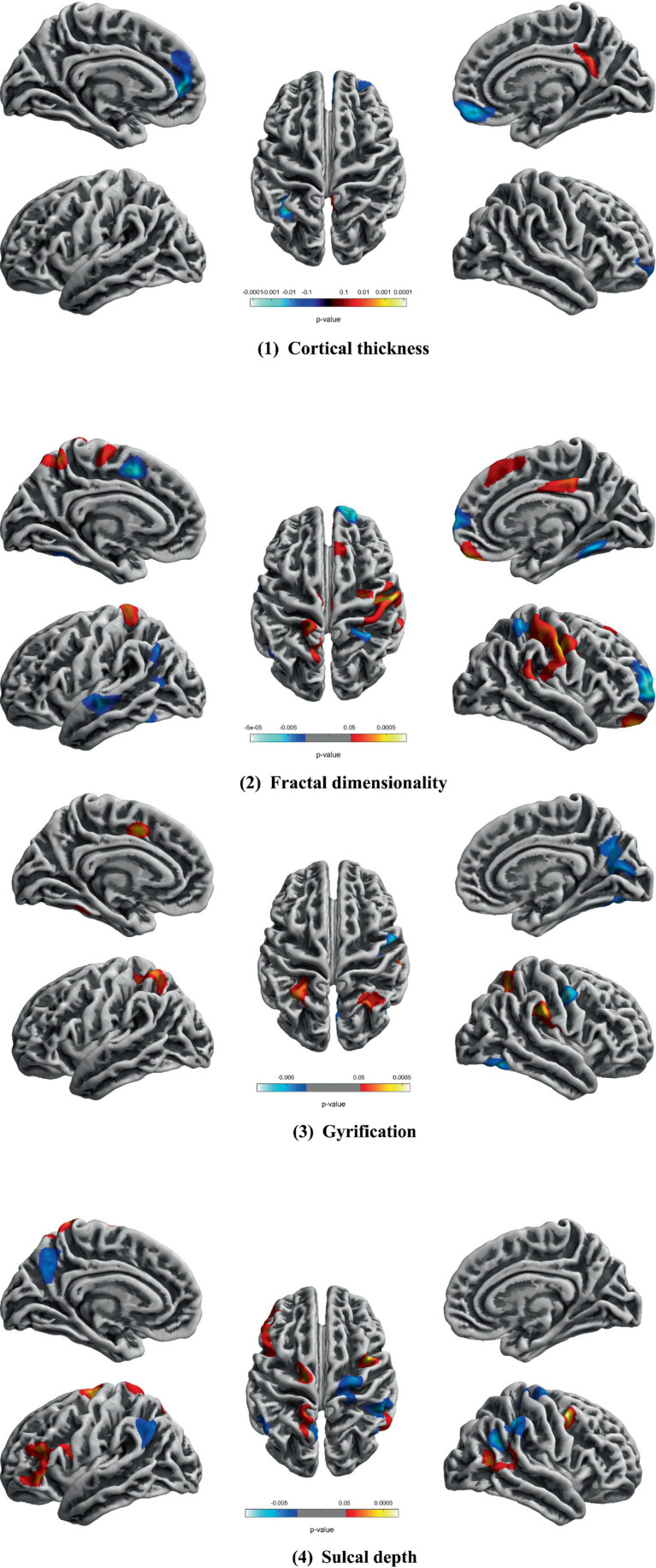
Cortical surface difference between FM vs. HC groups. Multiple comparison correction was performed using non-parametric permutations (*n* = 5,000) and threshold-free cluster enhancement (TFCE) *p* < 0.05. The threshold of cluster size is greater than 100 vertices. Red: FM group significantly greater than HC groups. Blue: FM group significantly less than HC groups. The left side in the figure indicates the left hemisphere, and the right side in the figure indicates the right hemisphere.

Compared to the HC group, the FM group had significantly greater CT in the right posterior cingulate cortex, but had significantly less CT in the left superior frontal gyrus, superior parietal lobule, caudal anterior cingulate cortex and the right medial orbitofrontal gyrus [see Figure 1 (1) and Table 2].

**Table 2:** Cortical surface difference between FM vs. HC groups.

Group differences	Regions	Cluster size	*p*-value
Thickness (left hemisphere)			
FM < HC	Superior frontal gyrus	259	0.000
	Superior parietal lobule	149	0.000
	Caudal anterior cingulate cortex	218	0.000
Thickness (right hemisphere)			
FM > HC	Posterior cingulate cortex	144	0.010
FM < HC	Medial orbitofrontal gyrus	152	0.004
Fractal dimensionality (left hemisphere)			
FM > HC	Precuneus	127	0.003
	Postcentral gyrus	123	0.002
FM < HC	Fusiform	226	0.001
	Superior temporal gyrus	289	0.003
	Superior frontal gyrus	186	0.001
	Inferior parietal lobule	154	0.006
Fractal dimensionality (right hemisphere)			
FM > HC	Postcentral gyrus	926	0.000
	Supramarginal gyrus	205	0.002
	Posterior cingulate cortex	219	0.005
	Superior frontal gyrus	201	0.027
	Precentral gyrus	115	0.001
FM < HC	Rostral middle frontal gyrus	191	0.000
	Superior parietal lobule	228	0.002
	Fusiform	154	0.003
Gyrification (left hemisphere)			
FM > HC	Superior parietal lobule	348	0.005
	Fusiform	125	0.016
	Superior frontal gyrus	146	0.001
Gyrification (right hemisphere)			
FM > HC	Superior parietal lobule	378	0.001
	Supramarginal gyrus	396	0.001
FM < HC	Precuneus	295	0.002
	Fusiform	180	0.002
	Precentral gyrus	207	0.000
Sulcal depth (left hemisphere)			
FM > HC	Pars triangularis gyrus	323	0.001
	Superior parietal lobule	172	0.006
FM < HC	Supramarginal gyrus	212	0.006
	Precuneus	322	0.006
Sulcal depth (right hemisphere)			
FM > HC	Caudal middle frontal gyrus	117	0.000
FM < HC	Precentral gyrus	312	0.003
	Supramarginal gyrus	372	0.001
	Inferior parietal lobule	205	0.000

Multiple comparison correction was performed using non-parametric permutations (*n =* 5,000) and threshold-free cluster enhancement (TFCE) p < 0.05. Cluster size refers to the number of vertices (surface mesh points) in the cluster. The threshold of cluster size is greater than 100 vertices. “FM > HC” means FM group significantly greater than HC groups. “FM < HC” means FM group significantly less than HC groups.

#### 3.2.2 FD

The FD results were bidirectional. Specifically, compared to the HC group, the FM group showed significantly greater FD in the following regions: the left precuneus, the right supramarginal, superior frontal, precentral gyri, posterior cingulate cortex, and the bilateral postcentral gyri. In contrast, the FM group showed significantly less FD in the left superior temporal, superior frontal gyri, inferior parietal lobule, the right superior parietal lobule, rostral middle frontal gyrus, and the bilateral fusiform gyri [see Figure 1 (2) and Table 2].

#### 3.2.3 GI

The GI results were also bidirectional. Compared to the HC group, the FM group showed significantly greater GI in the left fusiform, superior frontal gyri, the right supramarginal gyrus, and the bilateral superior parietal lobule. In contrast, the FM group showed significantly less GI in the right precuneus, fusiform and precentral gyri [see Figure 1 (3) and Table 2].

#### 3.2.4 SD

Compared to the HC group, the FM group had significantly greater SD in the left pars triangularis gyrus, superior parietal lobule, and the right caudal middle frontal gyrus. In contrast, the FM group had significantly less SD in the left precuneus, the right precentral gyrus, inferior parietal lobule, and the bilateral supramarginal gyri [see Figure 1 (4) and Table 2].

### 3.3 Correlation analysis

Exploratory correlation analyses (uncorrected *p*-values) revealed several associations between cortical metrics and clinical measures in the FM and HC groups. In the FM group, (1) The CT of the left caudal anterior cingulate cortex had significantly positive correlations to the HAMD and HAMA. (2) The FD of the right postcentral gyrus had significantly positive correlations to the HAMA and TAS, but had a significantly negative correlation to the MPQ. The FD of the right rostral middle frontal gyrus, and also the FD of the right superior frontal gyrus, had significantly positive correlations to the HAMD. (3) The GI of the left superior frontal gyrus had a significantly positive correlation to the TAS (see Table 3). No significant correlations between SD’s regions and clinical measures.

**Table 3:** Correlations between cortical surface morphometry and clinical measures.

Group	Cortical surface measures	Regions	Clinical measure	*r*	*p*
FM	CT	Left caudal anterior cingulate cortex	HAMD	0.353	0.044
		Left caudal anterior cingulate cortex	HAMA	0.433	0.012
	FD	Right postcentral gyrus	HAMA	0.366	0.036
		Right postcentral gyrus	TAS	0.419	0.015
		Right postcentral gyrus	MPQ	−0.366	0.036
		Right rostral middle frontal gyrus	HAMD	0.351	0.045
		Right superior frontal gyrus	HAMD	0.363	0.038
	GI	Left superior frontal gyrus	TAS	0.427	0.013
HC	CT	Left superior frontal gyrus	HAMD	−0.368	0.035
	FD	Left fusiform	HAMD	0.488	0.004
		Left inferior parietal lobule	TAS	−0.348	0.047
	GI	Left superior frontal gyrus	HAMD	0.526	0.002
		Right precuneus	HAMD	0.378	0.030
	SD	Right inferior parietal lobule	TAS	−0.374	0.032

In HC group, (1) The CT of the left superior frontal gyrus had a significantly negative correlation to the HAMD. (2) The FD of the left fusiform had a significantly positive correlation to the HAMD; the FD of the left inferior parietal lobule had a significantly negative correlation to the TAS. (3) The GI of the left superior frontal gyrus, and also the GI of the right precuneus, had the significantly correlations to the HAMD. (4) The SD of the right inferior parietal lobule had a significantly negative correlation to the TAS (see Table 3).

A complete list of all tested correlations with uncorrected *p*-values, including the non-significant ones, is provided in Supplementary Table S1 for the FM group and in Supplementary Table S2 for the HC group.

However, after applying Bonferroni correction for multiple comparisons (280 tests per group, corrected *α* = 0.00018), none of these correlations reached statistical significance. Under a more liberal FDR correction (*q* < 0.05), only a subset remained significant: in the FM group, the CT of the left caudal anterior cingulate cortex remained positively correlated with HAMD (*r* = 0.353, uncorrected *p* = 0.044, *q* = 0.028). In the HC group, the GI of the left superior frontal gyrus remained positively correlated with HAMD (*r* = 0.526, uncorrected *p* = 0.002, *q* = 0.028). Moreover, two additional correlations showed *q*-values between 0.05 and 0.10 (i.e., borderline under FDR): in the FM group, CT of the left caudal anterior cingulate cortex with HAMA (*r* = 0.433, uncorrected *p* = 0.012, *q* = 0.084) and FD of the right postcentral gyrus with TAS (*r* = 0.419, uncorrected *p* = 0.015, *q* = 0.090). These should be interpreted as exploratory trends only, not as statistically significant findings. All other correlations reported in Table 3 did not survive FDR correction and should be considered purely exploratory. Therefore, the primary interpretation of these correlation findings is that they are hypothesis-generating and require independent replication.

## 4 Discussion

The present study employed a comprehensive multi-parametric SBM approach to investigate cortical structural alterations in female patients with FM compared to age-matched female HC group. To our knowledge, this is the first study to simultaneously analyze CT, FD, GI and SD in the same FM cohort. The present results revealed widespread, bidirectional alterations across all four metrics, with each showing regionally specific changes. Furthermore, these structural differences were significantly correlated with clinical measures of pain, depression, anxiety, and alexithymia, underscoring the close relationship between brain structure and the multifaceted clinical presentation of FM.

### 4.1 Clinical characteristics

Consistent with the well-established clinical phenotype of fibromyalgia, the present study found that FM patients reported significantly higher levels of depression, anxiety, and alexithymia compared to healthy controls, as measured by the HAMD, HAMA, and TAS, respectively. These findings are in line with a large body of literature documenting high psychiatric comorbidity in FM, where the lifetime prevalence of major depressive disorder exceeds 50% and anxiety disorders affect up to one-third of patients (Galvez-Sanchez et al., 2019; Oliveria Neto et al., 2025; Rahangdale and Ferraro, 2025). The elevated TAS score further support the notion that FM is characterized not only by emotional distress but also by a specific deficit in identifying and describing one’s own emotions, which may exacerbate pain perception and impair adaptive coping mechanisms (Balducci et al., 2024).

In contrast, no significant group differences were observed for the FIQ or MPQ. The lack of difference on the FIQ—a measure of fibromyalgia impact—may reflect the fact that this questionnaire was designed to assess disease-specific functional impairment, and the HC group’s scores likely represent floor effects or non-specific responses rather than true disease impact. The absence of a significant MPQ difference is more surprising, as one might expect FM patients to report higher pain intensity. However, the MPQ scores in this sample were relatively low in both groups, which may be attributable to the specific characteristics of the public dataset used or to the possibility that the MPQ was administered as a measure of general pain quality rather than current pain intensity. Alternatively, this finding could reflect the heterogeneous nature of FM, where some patients experience more pronounced cognitive/affective symptoms than sensory pain at the time of assessment. Nevertheless, the lack of group difference on the MPQ does not diminish the clinical relevance of the FM diagnosis, as all patients met established diagnostic criteria (Balducci et al., 2024), and significant differences were found on multiple other clinically meaningful scales (HAMD, HAMA, TAS).

### 4.2 Multi-parametric cortical alterations in the FM group

In the FM group, cortical thinning was observed in the left superior frontal gyrus (SFG), superior parietal lobule (SPL), and caudal anterior cingulate cortex (cACC), and right medial orbitofrontal gyrus (mOFC), while greater CT was found in the right posterior cingulate cortex (PCC). The thinning in the left cACC and right mOFC—core limbic nodes involved in affective pain processing and emotional regulation (Jurado-Priego et al., 2024; Shackman et al., 2011)—aligns with previous reports of gray matter reductions in these regions (Oliveria Neto et al., 2025; Xin et al., 2023). Exploratorily, the positive correlation between CT in the left cACC and depression/anxiety score in the FM group indicates that relatively greater CT (i.e., less thinning) in this region is associated with more severe affective symptoms. Although counterintuitive, this may reflect a maladaptive structural response or a pre-existing vulnerability trait, though longitudinal data are needed to test these possibilities. Cortical thinning in the left SFG and SPL—key nodes of the default mode and frontoparietal networks—may correspond to the cognitive complaints (“fibro-fog”) commonly reported by FM patients (Kravitz and Katz, 2015). The greater CT in the right PCC, a central hub of the default mode network involved in self-referential thought (Azarias et al., 2025; Vatansever et al., 2018), may represent a structural correlate of previously reported functional hyperactivation and altered connectivity in this region (Ackermann et al., 2025; Stroman et al., 2025).

FD showed bidirectional alterations: greater FD in sensorimotor regions (bilateral postcentral gyri, right precentral gyrus) and less FD in visual association and temporal areas (bilateral fusiform, left superior temporal gyrus, right rostral middle frontal gyrus). The greater FD in the postcentral gyrus—the primary somatosensory cortex—is particularly compelling, as its hyper-reactivity is a hallmark of central sensitization in the FM group (Jurado-Priego et al., 2024). Correlation analyses revealed that greater FD in the right postcentral gyrus was positively correlated with anxiety (HAMA) and alexithymia (TAS) but negatively correlated with pain intensity (MPQ). The negative correlation with MPQ suggests an association between greater structural complexity and lower pain intensity, possibly reflecting more differentiated sensory processing (Chen et al., 2020). The positive correlation with alexithymia may indicate altered somatic signal processing, though this hypothesis requires direct testing with functional paradigms. Conversely, less FD in the bilateral fusiform and left superior temporal gyrus—regions critical for face/object recognition and social cognition (Harry et al., 2013; Thye et al., 2024)—suggests reduced structural complexity that may contribute to difficulties in processing emotional stimuli and social cues, consistent with functional findings in the FM group (Balducci et al., 2024).

GI also showed bidirectional changes: greater GI in the bilateral SPL, left SFG, and right supramarginal gyrus, and less GI in the right precuneus, fusiform, and precentral gyri. GI is largely established during neurodevelopment but can be influenced by later plastic changes (Li Y. et al., 2021). Greater GI in the SPL and SFG may represent a compensatory trait, whereas less GI in the right precuneus—a core default mode network hub—aligns with the pattern of reduced complexity observed in FD and may be a structural correlate of altered self-referential activity in the FM group (Stroman et al., 2025). The positive correlation between GI in the left SFG and alexithymia (TAS) further demonstrates an association between prefrontal cortical structure and emotional awareness.

Finally, SD alterations included greater depth in the left pars triangularis and right caudal middle frontal gyrus, and less depth in the bilateral supramarginal gyri, left precuneus, and right inferior parietal lobule (IPL). SD is influenced by underlying white matter tract tension and reflects network-level reorganization (Jin et al., 2018). The less SD in the bilateral supramarginal gyri and right IPL—core frontoparietal network regions involved in attention and body ownership—may indicate altered white matter integrity affecting communication efficiency and contributing to cognitive and sensory integration difficulties in the FM group.

### 4.3 Clinical correlations of structural alterations

The correlation analyses provide a crucial link between structural findings and clinical symptoms. Different metrics in different regions correlated with specific symptom domains, suggesting distributed network-level pathology. First, after FDR correction, depression (HAMD) was significantly correlated with CT in the cACC. Anxiety (HAMA) also showed a positive correlation with CT in the cACC, although this was borderline under FDR correction. Additionally, several exploratory uncorrected correlations were observed between affective symptoms and FD in the right prefrontal cortex (rostral middle frontal and superior frontal gyri; see Table 3 for uncorrected *p*-values), underscoring the potential role of fronto-cingulate circuits in the FM group’s high psychiatric comorbidity (Xin et al., 2023). Second, alexithymia (TAS) showed a borderline positive correlation with FD in the right postcentral gyrus under FDR correction. In addition, several exploratory uncorrected correlations were observed between alexithymia and other metrics, including GI in the left superior frontal gyrus and, in the HC group, FD and SD in parietal regions (see Table 3 for uncorrected *p*-values). This multi-focal pattern aligns with the view of alexithymia as a disorder of emotional awareness involving somatosensory, limbic, and prefrontal regions. Third, pain intensity (MPQ) showed a negative correlation with FD in the right postcentral gyrus, although this did not survive FDR correction. This exploratory finding suggests a possible correlational relationship between primary sensory cortex complexity and pain experience, though causal direction cannot be inferred and replication is needed.

### 4.4 Comparison with functional findings and limitations

The present structural findings are spatially consistent with the functional work of Balducci et al. (2024), who reported altered occipital and cingulate activation during emotional processing in the FM group. However, because we did not acquire functional data from the same individuals, we cannot directly link the present structural findings to their functional connectivity findings. Future multimodal studies combing SBM with resting-state and task-based fMRI in the same cohort are needed to directly investigate structure–function relationships in the FM.

Several limitations should be acknowledged. First, the cross-sectional design precludes causal inference. Longitudinal studies are needed to determine whether these structural alterations predate FM or result from chronic pain. Second, our sample was limited to females, so findings cannot be generalized to males with FM. Third, the original public dataset did not include systematic records of individual medication use and psychiatric comorbidity. While we used the HAMD and HAMA to quantify symptom severity, these scales do not substitute for structured diagnostic interviews. Therefore, the present study could not perform reliable covariate analyses to fully disentangle disease-specific effects from those of comorbidities or medications. We acknowledge this limitation and emphasize that the present findings represent a real-world FM sample with typical clinical heterogeneity. Residual confounding cannot be ruled out, and future medication-naïve, longitudinal studies with detailed psychiatric phenotyping are needed to confirm the specificity of the observed cortical alterations. Fourth, correlation analyses in Table 3 were not corrected for multiple comparisons; these findings should be considered exploratory and require independent replication. When applied Bonferroni correction, none of the reported associations survived. Under FDR correction, only a small subset remained significant or borderline. Therefore, these correlational findings should be considered strictly exploratory and hypothesis-generating rather than confirmatory. Future studies with pre-registered hypotheses are needed to validate these preliminary observations. Finally, the modest sample size (N = 33 per group) remains a limitation, though it is comparable to or larger than many previous SBM studies in FM. Future large-scale, prospective cohorts are needed to confirm the stability and generalizability of the present multi-parametric cortical phenotype.

## 5 Conclusion

In conclusion, this comprehensive surface-based morphometry study reveals that fibromyalgia is associated with widespread, multi-faceted alterations in cortical structure. By employing CT, FD, GI, and SD, we demonstrate that the neuroanatomical phenotype of FM consists of regionally specific patterns of both increases and decreases in thickness, complexity, folding, and sulcal geometry. These structural differences are prominently located in brain networks central to pain processing, emotion regulation, and self-referential cognition. Notably, the exploratory correlations between the structural alterations and clinical measures of depression, anxiety, alexithymia, and pain intensity underscore the clinical relevance of the observed cortical changes, although these findings require independent replication. The present findings provide a robust structural framework that complements existing functional models of FM and highlight key brain regions as potential targets for future hypothesis-driven research and therapeutic interventions.

## Data Availability

The datasets presented in this study can be found in online repositories. The names of the repository/repositories and accession number(s) can be found in the article/Supplementary material.

## Footnotes

1
https://openneuro.org/datasets/ds004144/versions/1.0.2

2
http://www.neuro.uni-jena.de/cat/

3
https://www.fil.ion.ucl.ac.uk/spm/software/spm12/
