# Supplementary material for: Alterations in cortical surface morphometry in fibromyalgia: a multi-parametric study
Source: Front Psychol. 2026 May 25;17:1813590. doi: 10.3389/fpsyg.2026.1813590 (PMC13243401; doi:10.3389/fpsyg.2026.1813590)
Supplement: Supplementary file 1 [file Table_1.docx]

**Supplementary Table S1.** Correlations between cortical surface morphometry and clinical measures in FM group.

| **Cortical surface measures** | **Clinical measure** | **Regions** | ***R*** | ***p*** |
| --- | --- | --- | --- | --- |
| CT | HAMD | Left caudal anterior cingulate cortex | 0.353^*^ | .044 |
|  |  | Right medial orbitofrontal gyrus | 0.104 | .565 |
|  |  | Right posterior cingulate cortex | 0.011 | .953 |
|  |  | Left superior frontal gyrus | 0.016 | .931 |
|  |  | Left superior parietal lobule | 0.007 | .971 |
|  | HAMA | Left caudal anterior cingulate cortex | 0.433^*^ | .012 |
|  |  | Right medial orbitofrontal gyrus | 0.071 | .694 |
|  |  | Right posterior cingulate cortex | 0.073 | .685 |
|  |  | Left superior frontal gyrus | -0.097 | .590 |
|  |  | Left superior parietal lobule | -0.223 | .212 |
|  | TAS | Left caudal anterior cingulate cortex | -0.061 | .734 |
|  |  | Right medial orbitofrontal gyrus | 0.002 | .991 |
|  |  | Right posterior cingulate cortex | -0.419^*^ | .015 |
|  |  | Left superior frontal gyrus | -0.276 | .120 |
|  |  | Left superior parietal lobule | -0.053 | .770 |
|  | FIQ | Left caudal anterior cingulate cortex | -0.160 | .372 |
|  |  | Right medial orbitofrontal gyrus | 0.142 | .431 |
|  |  | Right posterior cingulate cortex | 0.012 | .947 |
|  |  | Left superior frontal gyrus | 0.177 | .326 |
|  |  | Left superior parietal lobule | 0.170 | .345 |
|  | MPQ | Left caudal anterior cingulate cortex | 0.313 | .076 |
|  |  | Right medial orbitofrontal gyrus | 0.103 | .570 |
|  |  | Right posterior cingulate cortex | 0.123 | .495 |
|  |  | Left superior frontal gyrus | 0.023 | .897 |
|  |  | Left superior parietal lobule | 0.207 | .249 |
| FD | HAMD | Left fusiform | 0.100 | .579 |
|  |  | Right fusiform | 0.069 | .705 |
|  |  | Left inferior parietal lobule | 0.088 | .625 |
|  |  | Left postcentral gyrus | 0.042 | .818 |
|  |  | Right postcentral gyrus | 0.304 | .086 |
|  |  | Right posterior cingulate cortex | -0.170 | .345 |
|  |  | Right precentral gyrus | 0.022 | .902 |
|  |  | Left precuneus | -0.197 | .272 |
|  |  | Right rostral middle frontal gyrus | 0.351^*^ | .045 |
|  |  | Left superior frontal gyrus | 0.015 | .933 |
|  |  | Right superior frontal gyrus | 0.363^*^ | .038 |
|  |  | Right superior parietal lobule | -0.144 | .423 |
|  |  | Left superior temporal gyrus | 0.039 | .829 |
|  |  | Right supramarginal gyrus | 0.159 | .376 |
|  | HAMA | Left fusiform | 0.235 | .187 |
|  |  | Right fusiform | 0.053 | .770 |
|  |  | Left inferior parietal lobule | 0.130 | .471 |
|  |  | Left postcentral gyrus | 0.030 | .868 |
|  |  | Right postcentral gyrus | 0.366^*^ | .036 |
|  |  | Right posterior cingulate cortex | 0.075 | .677 |
|  |  | Right precentral gyrus | 0.085 | .639 |
|  |  | Left precuneus | -0.252 | .158 |
|  |  | Right rostral middle frontal gyrus | 0.166 | .356 |
|  |  | Left superior frontal gyrus | -0.007 | .970 |
|  |  | Right superior frontal gyrus | 0.323 | .067 |
|  |  | Right superior parietal lobule | -0.213 | .233 |
|  |  | Left superior temporal gyrus | -0.090 | .620 |
|  |  | Right supramarginal gyrus | 0.129 | .473 |
|  | TAS | Left fusiform | 0.256 | .150 |
|  |  | Right fusiform | 0.172 | .338 |
|  |  | Left inferior parietal lobule | -0.002 | .990 |
|  |  | Left postcentral gyrus | 0.118 | .511 |
|  |  | Right postcentral gyrus | 0.419^*^ | .015 |
|  |  | Right posterior cingulate cortex | 0.197 | .271 |
|  |  | Right precentral gyrus | -0.299 | .091 |
|  |  | Left precuneus | 0.224 | .209 |
|  |  | Right rostral middle frontal gyrus | -0.152 | .399 |
|  |  | Left superior frontal gyrus | 0.311 | .078 |
|  |  | Right superior frontal gyrus | 0.170 | .344 |
|  |  | Right superior parietal lobule | 0.137 | .448 |
|  |  | Left superior temporal gyrus | 0.030 | .867 |
|  |  | Right supramarginal gyrus | 0.287 | .078 |
|  | FIQ | Left fusiform | -0.193 | .283 |
|  |  | Right fusiform | 0.013 | .945 |
|  |  | Left inferior parietal lobule | -0.013 | .943 |
|  |  | Left postcentral gyrus | -0.027 | .882 |
|  |  | Right postcentral gyrus | 0.161 | .371 |
|  |  | Right posterior cingulate cortex | -0.32 | .070 |
|  |  | Right precentral gyrus | 0.031 | .863 |
|  |  | Left precuneus | 0.079 | .662 |
|  |  | Right rostral middle frontal gyrus | 0.287 | .105 |
|  |  | Left superior frontal gyrus | 0.109 | .547 |
|  |  | Right superior frontal gyrus | 0.136 | .450 |
|  |  | Right superior parietal lobule | -0.120 | .507 |
|  |  | Left superior temporal gyrus | -0.205 | .253 |
|  |  | Right supramarginal gyrus | -0.142 | .429 |
|  | MPQ | Left fusiform | 0.138 | .443 |
|  |  | Right fusiform | 0.108 | .548 |
|  |  | Left inferior parietal lobule | 0.192 | .283 |
|  |  | Left postcentral gyrus | -0.126 | .486 |
|  |  | Right postcentral gyrus | -0.366^*^ | .036 |
|  |  | Right posterior cingulate cortex | 0.008 | .965 |
|  |  | Right precentral gyrus | -0.055 | .759 |
|  |  | Left precuneus | -0.063 | .730 |
|  |  | Right rostral middle frontal gyrus | -0.003 | .987 |
|  |  | Left superior frontal gyrus | 0.034 | .853 |
|  |  | Right superior frontal gyrus | -0.215 | .230 |
|  |  | Right superior parietal lobule | 0.089 | .622 |
|  |  | Left superior temporal gyrus | -0.168 | .351 |
|  |  | Right supramarginal gyrus | 0.070 | .700 |
| GI | HAMD | Left fusiform | 0.230 | .198 |
|  |  | Right fusiform | 0.145 | .420 |
|  |  | Right precentral gyrus | -0.133 | .462 |
|  |  | Right precuneus | 0.083 | .646 |
|  |  | Left superior frontal gyrus | 0.138 | .444 |
|  |  | Left superior parietal lobule | -0.009 | .962 |
|  |  | Right superior parietal lobule | -0.177 | .324 |
|  |  | Right supramarginal gyrus | -0.142 | .431 |
|  | HAMA | Left fusiform | 0.274 | .123 |
|  |  | Right fusiform | 0.281 | .114 |
|  |  | Right precentral gyrus | -0.082 | .651 |
|  |  | Right precuneus | 0.063 | .726 |
|  |  | Left superior frontal gyrus | 0.069 | .702 |
|  |  | Left superior parietal lobule | -0.126 | .484 |
|  |  | Right superior parietal lobule | -0.244 | .170 |
|  |  | Right supramarginal gyrus | -0.156 | .386 |
|  | TAS | Left fusiform | 0.078 | .665 |
|  |  | Right fusiform | -0.030 | .870 |
|  |  | Right precentral gyrus | -0.169 | .348 |
|  |  | Right precuneus | 0.044 | .810 |
|  |  | Left superior frontal gyrus | 0.427^*^ | .013 |
|  |  | Left superior parietal lobule | -0.080 | .656 |
|  |  | Right superior parietal lobule | 0.121 | .504 |
|  |  | Right supramarginal gyrus | 0.211 | .239 |
|  | FIQ | Left fusiform | 0.210 | .241 |
|  |  | Right fusiform | 0.047 | .796 |
|  |  | Right precentral gyrus | 0.070 | .699 |
|  |  | Right precuneus | 0.036 | .843 |
|  |  | Left superior frontal gyrus | 0.131 | .469 |
|  |  | Left superior parietal lobule | 0.107 | .552 |
|  |  | Right superior parietal lobule | -0.196 | .274 |
|  |  | Right supramarginal gyrus | 0.138 | .442 |
|  | MPQ | Left fusiform | -0.023 | .897 |
|  |  | Right fusiform | -0.104 | .566 |
|  |  | Right precentral gyrus | 0.161 | .370 |
|  |  | Right precuneus | -0.270 | .129 |
|  |  | Left superior frontal gyrus | -0.204 | .254 |
|  |  | Left superior parietal lobule | -0.104 | .564 |
|  |  | Right superior parietal lobule | -0.082 | .651 |
|  |  | Right supramarginal gyrus | -0.070 | .698 |
| SD | HAMD | Right caudal middle frontal gyrus | -0.076 | .673 |
|  |  | Right inferior parietal lobule | -0.107 | .555 |
|  |  | Left parstriangularis | 0.092 | .610 |
|  |  | Right precentral gyrus | 0.178 | .322 |
|  |  | Left precuneus | -0.077 | .669 |
|  |  | Left superior parietal lobule | 0.005 | .979 |
|  |  | Left supramarginal gyrus | 0.192 | .284 |
|  |  | Right supramarginal gyrus | -0.137 | .448 |
|  | HAMA | Right caudal middle frontal gyrus | -0.001 | .995 |
|  |  | Right inferior parietal lobule | -0.085 | .638 |
|  |  | Left parstriangularis | 0.227 | .205 |
|  |  | Right precentral gyrus | 0.240 | .178 |
|  |  | Left precuneus | -0.164 | .361 |
|  |  | Left superior parietal lobule | 0.023 | .899 |
|  |  | Left supramarginal gyrus | 0.303 | .087 |
|  |  | Right supramarginal gyrus | 0.073 | .685 |
|  | TAS | Right caudal middle frontal gyrus | 0.014 | .938 |
|  |  | Right inferior parietal lobule | 0.241 | .176 |
|  |  | Left parstriangularis | 0.194 | .280 |
|  |  | Right precentral gyrus | 0.131 | .466 |
|  |  | Left precuneus | 0.190 | .289 |
|  |  | Left superior parietal lobule | 0.127 | .480 |
|  |  | Left supramarginal gyrus | 0.210 | .241 |
|  |  | Right supramarginal gyrus | 0.024 | .895 |
|  | FIQ | Right caudal middle frontal gyrus | -0.309 | .080 |
|  |  | Right inferior parietal lobule | -0.151 | .401 |
|  |  | Left parstriangularis | -0.128 | .479 |
|  |  | Right precentral gyrus | -0.221 | .217 |
|  |  | Left precuneus | -0.174 | .332 |
|  |  | Left superior parietal lobule | -0.109 | .547 |
|  |  | Left supramarginal gyrus | -0.036 | .844 |
|  |  | Right supramarginal gyrus | -0.227 | .205 |
|  | MPQ | Right caudal middle frontal gyrus | 0.112 | .534 |
|  |  | Right inferior parietal lobule | -0.041 | .821 |
|  |  | Left parstriangularis | -0.024 | .895 |
|  |  | Right precentral gyrus | 0.264 | .138 |
|  |  | Left precuneus | 0.216 | .228 |
|  |  | Left superior parietal lobule | -0.026 | .884 |
|  |  | Left supramarginal gyrus | -0.037 | .838 |
|  |  | Right supramarginal gyrus | 0.123 | .496 |

Note: ^*^*p* < .05. This complete list also includes correlations with uncorrected *p*-values, including the non-significant ones. CT: cortical

thickness; FD: fractal dimensionality; GI: gyrification; SD: sulcal depth; HAMD: Hamilton Depression Rating Scale; HAMA: Hamilton Anxiety Rating Scale; TAS: Toronto Alexithymia Scale; FIQ: Fibromyalgia Impact Questionnaire; MPQ: McGill Pain Questionnaire.

**Supplementary Table S2.** Correlations between cortical surface morphometry and clinical measures in healthy control group.

| **Cortical surface measures** | **Clinical measure** | **Regions** | ***R*** | ***p*** |
| --- | --- | --- | --- | --- |
| CT | HAMD | Left caudal anterior cingulate cortex | -0.056 | 0.758 |
|  |  | Right medial orbitofrontal gyrus | -0.265 | 0.137 |
|  |  | Right posterior cingulate cortex | -0.038 | 0.834 |
|  |  | Left superior frontal gyrus | -0.368^*^ | 0.035 |
|  |  | Left superior parietal lobule | -0.025 | 0.890 |
|  | HAMA | Left caudal anterior cingulate cortex | 0.040 | 0.825 |
|  |  | Right medial orbitofrontal gyrus | -0.033 | 0.855 |
|  |  | Right posterior cingulate cortex | -0.257 | 0.149 |
|  |  | Left superior frontal gyrus | -0.100 | 0.580 |
|  |  | Left superior parietal lobule | 0.073 | 0.685 |
|  | TAS | Left caudal anterior cingulate cortex | -0.247 | 0.165 |
|  |  | Right medial orbitofrontal gyrus | -0.125 | 0.489 |
|  |  | Right posterior cingulate cortex | -0.329 | 0.061 |
|  |  | Left superior frontal gyrus | -0.185 | 0.304 |
|  |  | Left superior parietal lobule | -0.088 | 0.625 |
|  | FIQ | Left caudal anterior cingulate cortex | -0.185 | 0.304 |
|  |  | Right medial orbitofrontal gyrus | -0.005 | 0.978 |
|  |  | Right posterior cingulate cortex | 0.054 | 0.764 |
|  |  | Left superior frontal gyrus | -0.048 | 0.792 |
|  |  | Left superior parietal lobule | -0.211 | 0.239 |
|  | MPQ | Left caudal anterior cingulate cortex | -0.100 | 0.579 |
|  |  | Right medial orbitofrontal gyrus | 0.005 | 0.978 |
|  |  | Right posterior cingulate cortex | 0.152 | 0.399 |
|  |  | Left superior frontal gyrus | 0.142 | 0.431 |
|  |  | Left superior parietal lobule | 0.296 | 0.094 |
| FD | HAMD | Left fusiform | 0.488^***^ | 0.004 |
|  |  | Right fusiform | 0.276 | .120 |
|  |  | Left inferior parietal lobule | 0.307 | .083 |
|  |  | Left postcentral gyrus | 0.267 | .133 |
|  |  | Right postcentral gyrus | 0.089 | .623 |
|  |  | Right posterior cingulate cortex | 0.117 | .518 |
|  |  | Right precentral gyrus | -0.053 | .770 |
|  |  | Left precuneus | 0.301 | .088 |
|  |  | Right rostral middle frontal gyrus | -0.100 | .581 |
|  |  | Left superior frontal gyrus | 0.161 | .370 |
|  |  | Right superior frontal gyrus | 0.094 | .603 |
|  |  | Right superior parietal lobule | 0.146 | .417 |
|  |  | Left superior temporal gyrus | 0.163 | .364 |
|  |  | Right supramarginal gyrus | 0.308 | .081 |
|  | HAMA | Left fusiform | 0.188 | .295 |
|  |  | Right fusiform | 0.097 | .590 |
|  |  | Left inferior parietal lobule | -0.078 | .666 |
|  |  | Left postcentral gyrus | 0.095 | .600 |
|  |  | Right postcentral gyrus | 0.120 | .504 |
|  |  | Right posterior cingulate cortex | 0.057 | .751 |
|  |  | Right precentral gyrus | -0.009 | .962 |
|  |  | Left precuneus | 0.208 | .245 |
|  |  | Right rostral middle frontal gyrus | 0.119 | .508 |
|  |  | Left superior frontal gyrus | 0.243 | .174 |
|  |  | Right superior frontal gyrus | 0.169 | .348 |
|  |  | Right superior parietal lobule | 0.043 | .814 |
|  |  | Left superior temporal gyrus | 0.225 | .207 |
|  |  | Right supramarginal gyrus | 0.167 | .353 |
|  | TAS | Left fusiform | -0.038 | .834 |
|  |  | Right fusiform | -0.085 | .638 |
|  |  | Left inferior parietal lobule | -0.348^*^ | .047 |
|  |  | Left postcentral gyrus | 0.326 | .064 |
|  |  | Right postcentral gyrus | 0.040 | .824 |
|  |  | Right posterior cingulate cortex | 0.004 | .982 |
|  |  | Right precentral gyrus | 0.062 | .734 |
|  |  | Left precuneus | 0.153 | .395 |
|  |  | Right rostral middle frontal gyrus | 0.233 | .192 |
|  |  | Left superior frontal gyrus | 0.037 | .838 |
|  |  | Right superior frontal gyrus | 0.305 | .084 |
|  |  | Right superior parietal lobule | -0.246 | .167 |
|  |  | Left superior temporal gyrus | -0.086 | .634 |
|  |  | Right supramarginal gyrus | -0.115 | .524 |
|  | FIQ | Left fusiform | -0.086 | .634 |
|  |  | Right fusiform | -0.111 | .537 |
|  |  | Left inferior parietal lobule | -0.187 | .298 |
|  |  | Left postcentral gyrus | -0.150 | .405 |
|  |  | Right postcentral gyrus | -0.180 | .315 |
|  |  | Right posterior cingulate cortex | 0.187 | .296 |
|  |  | Right precentral gyrus | 0.074 | .684 |
|  |  | Left precuneus | -0.118 | .532 |
|  |  | Right rostral middle frontal gyrus | 0.156 | .386 |
|  |  | Left superior frontal gyrus | -0.231 | .197 |
|  |  | Right superior frontal gyrus | -0.016 | .927 |
|  |  | Right superior parietal lobule | 0.213 | .234 |
|  |  | Left superior temporal gyrus | 0.222 | .215 |
|  |  | Right supramarginal gyrus | 0.079 | .662 |
|  | MPQ | Left fusiform | -0.039 | .831 |
|  |  | Right fusiform | 0.236 | .187 |
|  |  | Left inferior parietal lobule | 0.044 | .808 |
|  |  | Left postcentral gyrus | 0.175 | .330 |
|  |  | Right postcentral gyrus | -0.176 | .326 |
|  |  | Right posterior cingulate cortex | -0.237 | .185 |
|  |  | Right precentral gyrus | -0.124 | .490 |
|  |  | Left precuneus | 0.098 | .587 |
|  |  | Right rostral middle frontal gyrus | 0.068 | .707 |
|  |  | Left superior frontal gyrus | -0.048 | .789 |
|  |  | Right superior frontal gyrus | -0.120 | .507 |
|  |  | Right superior parietal lobule | -0.221 | .296 |
|  |  | Left superior temporal gyrus | 0.011 | .950 |
|  |  | Right supramarginal gyrus | -0.128 | .478 |
| GI | HAMD | Left fusiform | 0.025 | .891 |
|  |  | Right fusiform | 0.197 | .272 |
|  |  | Right precentral gyrus | 0.062 | .734 |
|  |  | Right precuneus | 0.378^*^ | .030 |
|  |  | Left superior frontal gyrus | 0.526^***^ | .002 |
|  |  | Left superior parietal lobule | 0.077 | .668 |
|  |  | Right superior parietal lobule | -0.019 | .918 |
|  |  | Right supramarginal gyrus | 0.230 | .198 |
|  | HAMA | Left fusiform | -0.070 | .700 |
|  |  | Right fusiform | -0.016 | .930 |
|  |  | Right precentral gyrus | 0.108 | .548 |
|  |  | Right precuneus | 0.158 | .380 |
|  |  | Left superior frontal gyrus | 0.336 | .056 |
|  |  | Left superior parietal lobule | 0.003 | .988 |
|  |  | Right superior parietal lobule | -0.039 | .831 |
|  |  | Right supramarginal gyrus | 0.037 | .837 |
|  | TAS | Left fusiform | -0.013 | .944 |
|  |  | Right fusiform | 0.149 | .409 |
|  |  | Right precentral gyrus | 0.237 | .184 |
|  |  | Right precuneus | 0.196 | .274 |
|  |  | Left superior frontal gyrus | 0.213 | .235 |
|  |  | Left superior parietal lobule | 0.044 | .806 |
|  |  | Right superior parietal lobule | 0.023 | .897 |
|  |  | Right supramarginal gyrus | 0.140 | .439 |
|  | FIQ | Left fusiform | 0.136 | .450 |
|  |  | Right fusiform | 0.124 | .493 |
|  |  | Right precentral gyrus | 0.133 | .459 |
|  |  | Right precuneus | -0.113 | .531 |
|  |  | Left superior frontal gyrus | -0.048 | .789 |
|  |  | Left superior parietal lobule | -0.163 | .364 |
|  |  | Right superior parietal lobule | -0.134 | .458 |
|  |  | Right supramarginal gyrus | -0.105 | .562 |
|  | MPQ | Left fusiform | -0.050 | .781 |
|  |  | Right fusiform | -0.222 | .214 |
|  |  | Right precentral gyrus | -0.180 | .309 |
|  |  | Right precuneus | -0.340 | .053 |
|  |  | Left superior frontal gyrus | -0.315 | .074 |
|  |  | Left superior parietal lobule | 0.002 | .993 |
|  |  | Right superior parietal lobule | -0.060 | .741 |
|  |  | Right supramarginal gyrus | -0.093 | .606 |
| SD | HAMD | Right caudal middle frontal gyrus | 0.127 | .483 |
|  |  | Right inferior parietal lobule | 0.310 | .079 |
|  |  | Left parstriangularis | 0.078 | .665 |
|  |  | Right precentral gyrus | 0.278 | .117 |
|  |  | Left precuneus | 0.227 | .204 |
|  |  | Left superior parietal lobule | -0.232 | .195 |
|  |  | Left supramarginal gyrus | -0.057 | .752 |
|  |  | Right supramarginal gyrus | 0.264 | .138 |
|  | HAMA | Right caudal middle frontal gyrus | 0.009 | .959 |
|  |  | Right inferior parietal lobule | 0.140 | .438 |
|  |  | Left parstriangularis | -0.012 | .949 |
|  |  | Right precentral gyrus | 0.252 | .158 |
|  |  | Left precuneus | 0.178 | .321 |
|  |  | Left superior parietal lobule | -0.052 | .776 |
|  |  | Left supramarginal gyrus | -0.244 | .171 |
|  |  | Right supramarginal gyrus | 0.190 | .291 |
|  | TAS | Right caudal middle frontal gyrus | -0.327 | .063 |
|  |  | Right inferior parietal lobule | -0.374 | .032 |
|  |  | Left parstriangularis | -0.214 | .232 |
|  |  | Right precentral gyrus | -0.041 | .821 |
|  |  | Left precuneus | 0.102 | .573 |
|  |  | Left superior parietal lobule | -0.038 | .834 |
|  |  | Left supramarginal gyrus | -0.139 | .441 |
|  |  | Right supramarginal gyrus | 0.136 | .450 |
|  | FIQ | Right caudal middle frontal gyrus | 0.032 | .859 |
|  |  | Right inferior parietal lobule | 0.146 | .419 |
|  |  | Left parstriangularis | -0.005 | .977 |
|  |  | Right precentral gyrus | -0.185 | .303 |
|  |  | Left precuneus | -0.321 | .068 |
|  |  | Left superior parietal lobule | 0.087 | .629 |
|  |  | Left supramarginal gyrus | 0.208 | .246 |
|  |  | Right supramarginal gyrus | 0.045 | .805 |
|  | MPQ | Right caudal middle frontal gyrus | 0.087 | .630 |
|  |  | Right inferior parietal lobule | -0.086 | .632 |
|  |  | Left parstriangularis | -0.094 | .604 |
|  |  | Right precentral gyrus | 0.042 | .816 |
|  |  | Left precuneus | -0.091 | .613 |
|  |  | Left superior parietal lobule | 0.136 | .450 |
|  |  | Left supramarginal gyrus | -0.166 | .355 |
|  |  | Right supramarginal gyrus | -0.175 | .331 |

Note: ^*^*p* < .05, ^***^*p* < .005. This complete list also includes correlations with uncorrected *p*-values, including the non-significant ones. CT: cortical thickness; FD: fractal dimensionality; GI: gyrification; SD: sulcal depth; HAMD: Hamilton Depression Rating Scale; HAMA: Hamilton Anxiety Rating Scale; TAS: Toronto Alexithymia Scale; FIQ: Fibromyalgia Impact Questionnaire; MPQ: McGill Pain Questionnaire.
